# Preoperative vena cava filter placement in recurrent cerebral fat embolism following traumatic multiple fractures

**DOI:** 10.1186/s13049-021-00906-1

**Published:** 2021-06-30

**Authors:** Silvia Di Bari, Marcello Bisulli, Emanuele Russo, Luca Bissoni, Costanza Martino, Luigi Branca Vergano, Domenico Pietro Santonastaso, Vito Marco Ranieri, Vanni Agnoletti

**Affiliations:** 1 Anesthesia and Intensive Care Department, Alma Mater Studiorum - Università di Bologna, IRCCS Azienda Ospedaliero-Universitaria di Bologna, Via Giuseppe Massarenti, 9, 40138 Bologna, Italy; 2 Interventional Radiology Department, AUSL Romagna Trauma Center “Maurizio Bufalini” Hospital, Viale Ghirotti 286, 47521 Cesena, Italy; 3 Anesthesia and Intensive Care Department, AUSL Romagna Trauma Center “Maurizio Bufalini” Hospital, Viale Ghirotti 286, 47521 Cesena, Italy; 4 Orthopedics and Traumatology Department, AUSL Romagna Trauma Center “Maurizio Bufalini” Hospital, Viale Ghirotti 286, 47521 Cesena, Italy

Dear Editor,

we read with great interest the study by Vetrugno et al. [1] where they provided a systematic review of published case reports of fat embolism syndrome (FES) following traumatic bone fractures. The authors found that FES is most frequent in young men and following multiple leg fractures. Remarkably, neither specific treatment of FES nor prevention guidelines exist, with supportive care being the only possible measure. They also reported how heparin and corticosteroids seemed to be beneficial but studies showed conflicting results.

We propose a brief case to suggest taking Inferior Vena Cava Filter (IVCF) placement into account as a prevention tool for recurrent fat embolism (FE) in selected patients. A 16-years old male was involved into a motor-scooter accident reporting right femoral shaft fracture and left tibia shaft fracture. External fixation was provided as a bridge treatment to surgery. A few hours later he suddenly developed respiratory failure and coma and was admitted to our Intensive Care Unit (ICU) for supportive care. Cerebral Computed Tomography (CT) was normal; total body CT scan with contrast excluded pulmonary thromboembolism (PTE) and showed diffuse interstitial and alveolar edema; electroencephalogram identified status epilepticus for which antiepileptic drugs were started. Cerebral magnetic resonance imaging revealed a “starfield” pattern compatible with the diagnosis of cerebral fat embolism. Patent foramen ovale was excluded. He was treated conservatively with mechanical ventilation with gradual improvement of his respiratory status, whereas neurologic impairment needed prolonged sedation. During his stay in the ICU he underwent sudden unexplained respiratory distress episodes, suggesting that embolization was possibly recurring. When stable, on day 7 of hospitalization, the patient was scheduled for surgical treatment. An IVCF was placed the day prior to surgery with the aim of stopping more fat emboli from spreading during intramedullary nailing of femur and tibia (Fig. 1). The operation was performed without peri-operative complications and the IVCF was removed 24 h later. The patient is currently recovering.

**Fig. 1:**
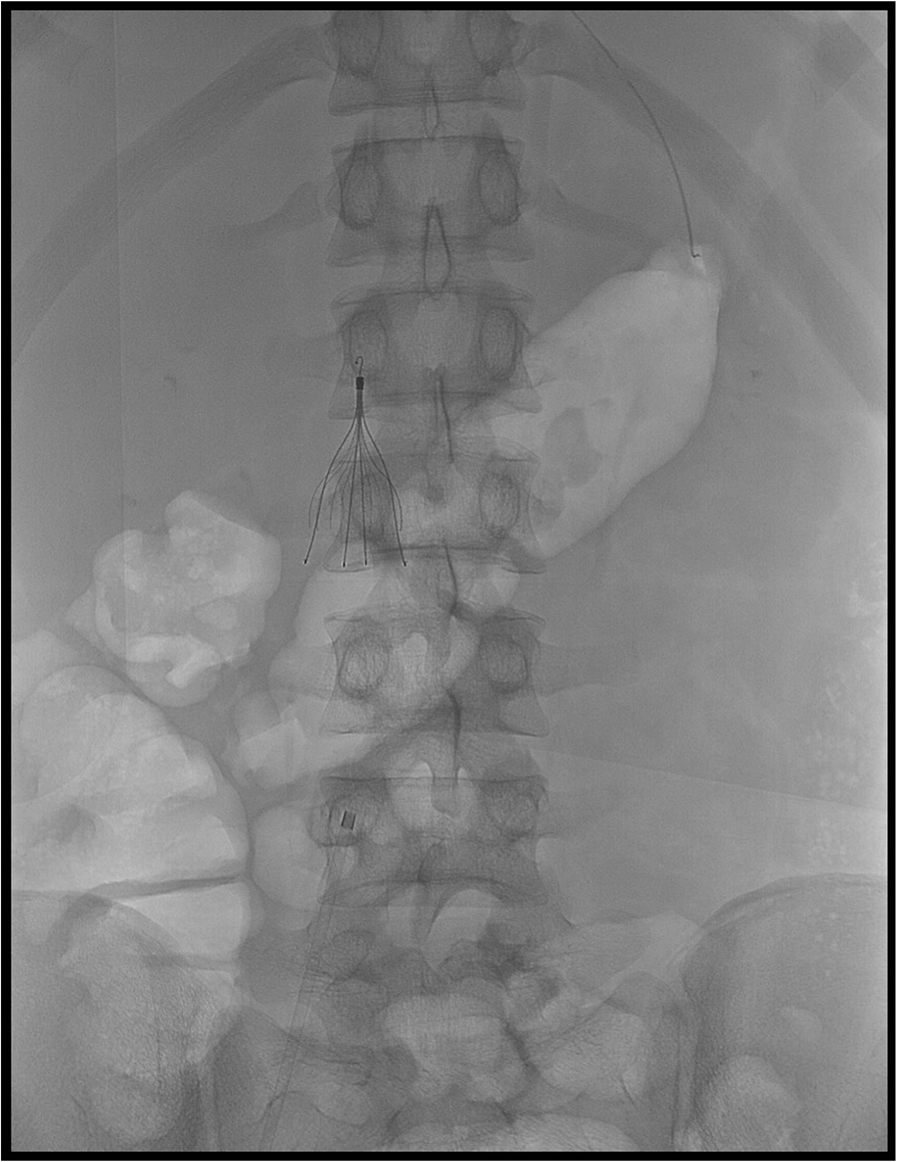
An inferior vena cava filter (IVCF) was placed prior to surgical stabilization of multiple traumatic fractures to prevent recurrency of fat embolism

Although IVCF insertion is discouraged for primary prevention of PTE in both orthopaedic surgery [2] and major trauma settings [3], all societies agree that it is warranted when deep vein thrombosis is present if anticoagulation is contraindicated or has failed [4]. Similarly, IVCF has been placed as FE prevention in three cases where CT imaging identified macroscopic fat emboli in large veins in traumatic patients, whether symptomatic or not, as reported by Burr et al. [5]. However, to our knowledge, no previous positioning of IVCF to specifically prevent fat embolization prior to orthopaedic stabilization in established FES has been reported.

## Conclusions

Despite scarce evidence, we reckon that IVCF could improve prognosis in selected patients with FES, especially those presenting high risk factors, after accurately weighing risks and benefits. This measure may also help to earlier stabilize the patient so that definitive surgical treatment of the fractures is promptly provided.

In conclusion, the study by Vetrugno et al. shed light on risk factors related to FES. However, further research is necessary to identify the best treatment and prevention strategies. Structured studies and prospective evaluation are needed to establish the effective benefits of IVC filter placement in similar cases.

## Data Availability

Data sharing not applicable to this article as no datasets were generated or analysed during the current study.

## Abbreviations

FES: Fat embolism syndrome
IVCF: Inferior Vena Cava Filter
FE: Fat embolism
ICU: Intensive Care Unit
CT: Computed Tomography
PTE: Pulmonary thromboembolism

## References

[1] Vetrugno L, Bignami E, Deana C, Bassi F, Vargas M, Orsaria M (2021). Cerebral fat embolism after traumatic bone fractures: a structured literature review and analysis of published case reports. Scand J Trauma Resusc Emerg Med.

[2] Falck-Ytter Y, Francis CW, Johanson NA, Curley C, Dahl OE, Schulman S (2012). Prevention of VTE in orthopedic surgery patients. Antithrombotic therapy and prevention of thrombosis, 9th ed: American College of Chest Physicians evidence-based clinical practice guidelines. Chest..

[3] Elkbuli A, Ehrhardt JD, Kinslow K, McKenney M (2021). Prophylactic inferior vena cava filters: outcomes in severely injured trauma patients. Am Surg.

[4] Li X, Haddadin I, McLennan G, Farivar B, Staub D, Beck A (2020). Inferior vena cava filter - comprehensive overview of current indications, techniques, complications and retrieval rates. Vasa..

[5] Burr T, Chaudhry H, Zhang C, Vasilopoulos V, Allam E (2020). Fat embolism in the popliteal vein detected on CT: case report and review of the literature. Radiol Case Rep.

